# Technique of vessel-skeletonized parenchyma-sparing hepatectomy for the oncological treatment of bilobar colorectal liver metastases

**DOI:** 10.1007/s00423-021-02373-9

**Published:** 2021-11-27

**Authors:** Yuzo Umeda, Takeshi Nagasaka, Kosei Takagi, Ryuichi Yoshida, Kazuhiro Yoshida, Tomokazu Fuji, Tatsuo Matsuda, Kazuya Yasui, Kenjiro Kumano, Hiroki Sato, Takahito Yagi, Toshiyoshi Fujiwara

**Affiliations:** 1 Department of Gastroenterological Surgery, Okayama University Graduate School of Medicine, Dentistry and Pharmaceutical Sciences, 2-5-1 Shikata-cho, Okayama City, Okayama 700-8558 Japan; 2 Department of Clinical Oncology, Kawasaki Medical School, Kurashiki, Okayama 701-0192 Japan

**Keywords:** Colorectal cancer, Hepatectomy, Liver metastasis, Parenchyma-sparing, Vessel skeletonization

## Abstract

**Background:**

To aid in the oncological management of multiple bilobar colorectal liver metastases (CRLMs), we describe a new surgical procedure, VEssel-Skeletonized PArenchyma-sparing Hepatectomy (VESPAH).

**Study design:**

Of 152 patients with CRLMs treated with hepatectomy, 33 patients had multiple bilobar liver metastases (≥8 liver metastases); their surgical procedures and clinical outcomes were retrospectively summarized and compared between those who underwent VESPAH and those who underwent major hepatectomy (Major Hx).

**Results:**

Of the 33 patients, 20 patients were resected by VESPAH (the VESPAH group) and 13 patients by major hepatectomy (Major Hx group). The median number of CRLMs was 13 (range, 8–53) in the VESPAH group and 10 (range, 8–41) in the Major Hx group (*P*=0.511). No operative mortality nor severe morbidity was observed in either group. The VESPAH group showed earlier recovery of remnant liver function after surgery than the Major Hx group; the incidence of grade B/C post hepatectomy liver failure was 5% in the VESPAH group and 38% in the Major Hx group, *P*=0.048). Intrahepatic tumor recurrence was confirmed in 14 (70%) and 7 (54%) patients in the VESPAH and Major Hx groups, respectively (*P*=0.416). There was no significant difference in median overall survival (OS) after hepatectomy between the two groups; the median OS was 47 months in the VESPAH group and 33 months in the Major Hx group (*P*=0.481). The VESPAH group showed the higher induction rate of adjuvant chemotherapy within 2 months after surgery (*P*=0.002) and total number of repeat hepatectomy for intrahepatic recurrence (*P*=0.060) than the Major Hx group.

**Conclusions:**

VESPAH enables us to clear surgical navigation by hepatic vessel skeletonization and may enhance patient tolerability of not only adjuvant chemotherapy but also repeat hepatectomies during the patients’ lifetimes.

**Supplementary Information:**

The online version contains supplementary material available at 10.1007/s00423-021-02373-9.

## Introduction

The liver is a common target for colorectal cancer (CRC) metastasis, the progression of which determines a patient’s outcome. At the time of diagnosis, colorectal liver metastases (CRLMs) are often deemed unresectable due to advanced progression, e.g., cases where metastases have already spread to particularly multiple bilobar lesions segments or have deeply invaded major blood vessels [1–4].

Approximately 45% of CRC patients with CRLMs require extended resection with sufficient surgical margins [5]. Although this surgical approach is highly important, extended liver resection may result in postoperative liver failure. This can be avoided by preserving more than 25% of the normal liver volume or more than 40% of the chemotherapy-damaged liver volume [6–8].

Several strategies have been developed to improve extensive hepatectomy in order to maximize the size of the remnant liver; these include portal vein embolization, two-stage hepatectomy (TSH), and associating liver partition and portal vein ligation for staged hepatectomy (ALPPS) [9–11]. The efficacy of these procedures has expanded the resection criteria to include previously unresectable CRLMs. However, these procedures still have limitations that need to be addressed. Indeed, approximately one-fourth of patients who are managed by TSH cannot undergo a second hepatectomy because of tumor progression or insufficient hypertrophy of the remnant liver after the first surgical procedure [12]. In contrast, ALPPS is designed to address the limitations of TSH by promoting rapid growth of the future remnant liver in a short timeframe, but this procedure is also associated with high morbidity and mortality rates [13]. The concerns surrounding TSH and ALPPS are mainly due to the difficulty in achieving oncological control of CRC during interval periods.

In contradiction of major hepatectomy (Major Hx), which involves systematic anatomical hepatic resection, the surgical procedure known as parenchyma-sparing hepatectomy aims to spare a certain future remnant liver volume [14–17]. Parenchyma-sparing hepatectomy is a surgical philosophy that utilizes limited nonanatomical liver resection to minimize surgical stress and operative risk. To manage and cure patients with multiple CRLMs oncologically, a surgical procedure based on the concept of parenchyma-sparing hepatectomy is needed to remove all liver metastases while preserving a sufficient future remnant liver volume.

Herein, we describe a new surgical procedure, named VEssel-Skeletonized PArenchyma-sparing Hepatectomy (VESPAH), for the management of patients with CRLMs. This technique can be adopted for CRC patients with multiple metastases located in the deep parenchyma or adjacent to major vessels and can achieve oncological tumor detachment to disrupt the invasive relationship between metastatic tumors and intrahepatic vessels. The top priority of VESPAH is how to minimize the resection volume and clean up multiple tumors efficiently. In addition, intrahepatic vessels should be preserved as much as possible in order to maintain good residual liver function, to increase the chance and variety of future repeat hepatectomy. To achieve these goals, tumor detachment from the main vascular structure is acceptable, and exposure of the liver skeleton can guide liver resection to ensure resection of deep small lesions.

## Materials and methods

### Study subjects

**Fig. 1:**
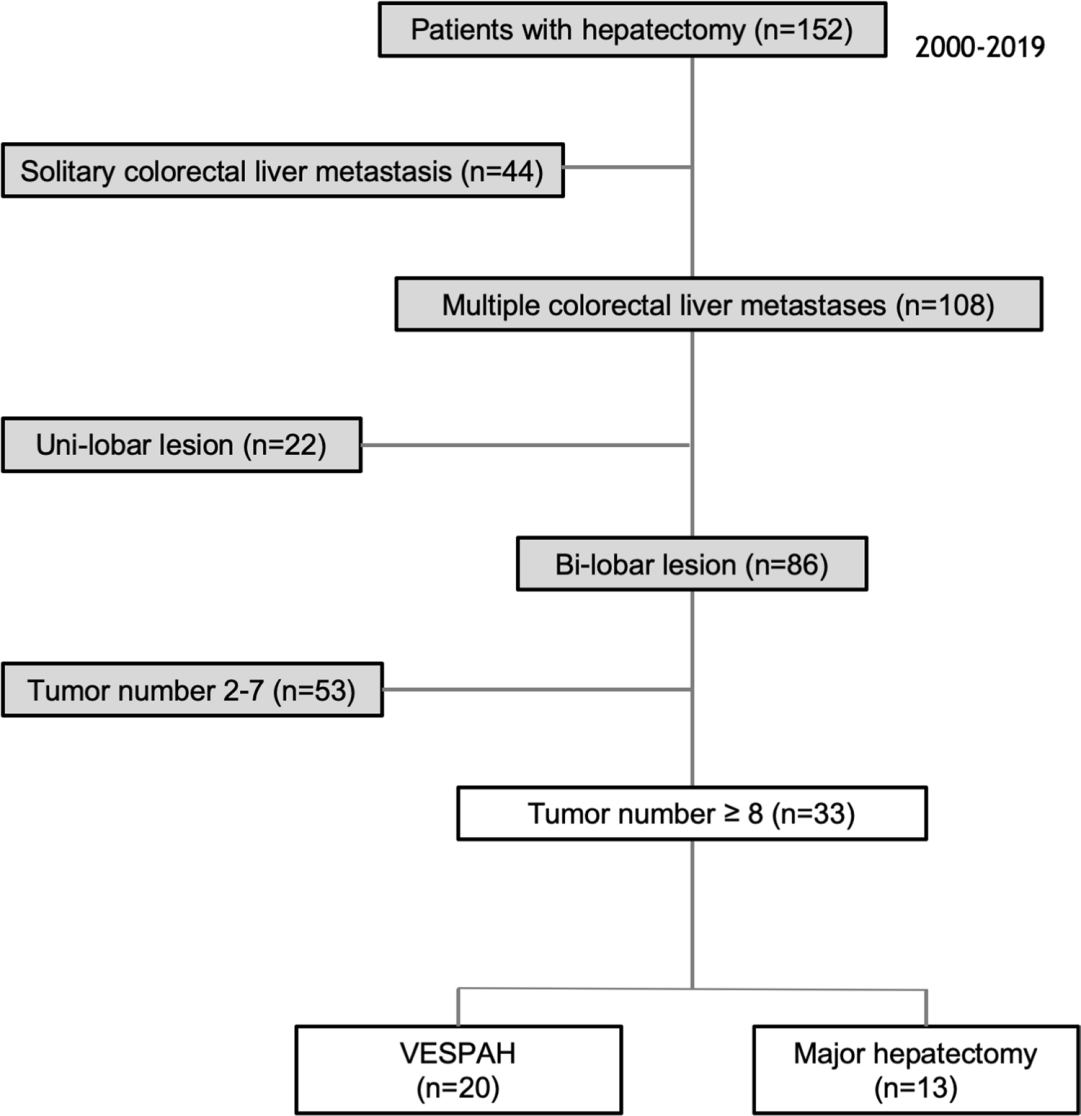
STROBE diagram of the patient cohort

This study retrospectively reviewed a cohort of 152 CRC patients with CRLMs who had achieved pathologically confirmed curative resection after having undergone hepatectomy between August 2000 and January 2019 at Okayama University Hospital (Figure 1).

There was no case treated by ablative procedure. The following demographic and clinical data were reviewed through medical records: gender, age at first hepatectomy, lymph node metastasis, genetic profiles, and location of the primary lesion. Clinical data of CRLMs was summarized in size, number, localization, contact with major hepatic veins, extrahepatic spread, information of pre- and postoperative chemotherapy, and preoperative risk assessed with a nomogram by Beppu T et al. (Beppu’s nomogram) and the Genetic And Morphological Evaluation (GAME) score [18, 19]. Beppu’s nomogram and GAME score are associated with postoperative recurrence risk and survival. Postoperative bile leakage and morbidity were assessed based on the definitions of the International Study Group of Liver Surgery [20] and Clavien-Dindo classification [21]. In the pathological examination of the resected specimens, a margin width ≥ 1 mm was defined as R0 resection, and a margin width < 1 mm was defined as R1 resection [22]. R1 status was categorized as R1-Par for tumor exposed to parenchyma or detached from main Glissonean pedicles, and R1-Vas for tumor detached from hepatic vein trunk.

In this study, we focused on patients with 8 or more bilobar CRLMs because of definitely considered to be unresectable. Among our initial cohort of 152 CRC patients with CRLMs, a total of 33 patients possessed 8 or more bilobar CRLMs. The 33 patients were classified into either the VESPAH or Major Hx group by their treated surgical procedure clearly indicated in the medical record; 13 patients were classified into the Major Hx group, and the remaining 20 patients were into the VESPAH group.

All 33 patients received preoperative chemotherapies, including doublet cytotoxic regimen; e. g., fluoropyrimidine (fluorouracil or capecitabine) plus oxaliplatin or irinotecan, triplet cytotoxic regimen; e. g., fluoropyrimidine (fluorouracil) plus oxaliplatin plus irinotecan, or hepatic artery infusion of fluoropyrimidine (fluorouracil). Target argents, anti-VEGF antibodies (bevacizumab or ramucirumab) or anti-EGFR antibodies (cetuximab or panitumumab) were added to the doublet cytotoxic regimens or the triplet cytotoxic regimen according to *RAS* mutational status and the preference of the patient.

### Terminology

The liver anatomy and resection terminology in this paper is based on the Brisbane classification [23]. Liver resections consisting of at least three adjacent liver segments of resection, such as left or right hemihepatectomy (Hr2) and trisectionectomy (Hr3), were defined as major hepatectomy.

### Operative technique of VEssel-Skeletonized PArenchyma-sparing Hepatectomy (VESPAH)

**Fig. 2:**
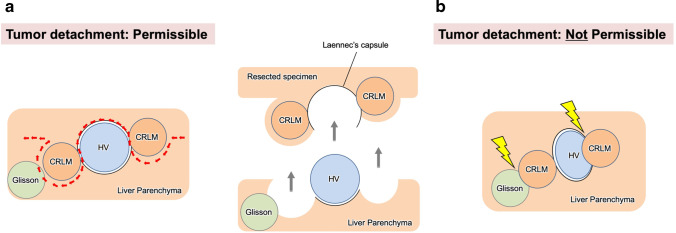
Permissibility of tumor detachment in vessel-skeletonized parenchyma-sparing hepatectomy. **a** A case in which tumor detachment is permissible. The liver transection line may be winding and include tumors in the liver parenchyma. Colorectal liver metastases can be detached with Laennec’s capsule from both the hepatic vein and/or the Glissonean pedicle. As a result, the intrahepatic structures are skeletonized and exposed to the polygonal cut surface of the liver. A red dotted line represents a liver transection line. **b** A case in which tumor detachment is not permissible. Tumors that invade the Glissonean pedicles with bile duct dilatation or that invade hepatic veins with wide adhesions or stenotic deformities cannot be detached

Oncologically, it is possible to achieve tumor detachment from just the outer layer of the hepatic vessels. VEssel-Skeletonized PArenchyma-sparing Hepatectomy (VESPAH) permits tumor detachment from the outer layer of the hepatic vessels, even in cases where space between a CRLM and a hepatic vessel is extremely narrow. On the other hand, tumor detachment is not recommended in cases where a metastatic tumor is widely adhered to a hepatic vein or if the radiological findings demonstrate deformed and/or stenotic hepatic vein. In contrast to hepatic veins, Glissonean pedicles can be exposed at the cut surface of the liver and preserved as a liver-skeleton (Figure 2a). However, in cases where metastatic lesions invade the Glissonean pedicles, the lesions should not be detached from the Glissonean pedicles (Figure 2b). In particular, in cases of intrahepatic peripheral bile duct dilatation adjacent to liver metastases, the minimum anatomical area perfused by the Glissonean pedicle should be resected along with the pedicle division on the proximal side of its tumor invasion. In other words, extensive invasion to the first/second-order of Glissonean pedicles requires hemi-hepatectomy or sectionectomy, while Glissonean pedicles beyond the third-order require segmentectomy/sub-segmentectomy. And in other aspect, Vessel-Skeletonization can contribute to navigation to small multiple lesions located in the deep liver parenchyma and to gaining surgical margin. Parenchyma-sparing hepatectomy allowing R1 resection is firstly reported as R1 vascular resection by Italian group [24, 25]. In this analysis, detached from main Glissonean pedicles was defined as R1-par. And, in other aspect, exposure of the liver skeleton can guide liver resection to ensure resection of deep small lesions. These are the ways in which VESPAH is both similar and different from their R1 vascular resection.

Regarding the technical details of VESPAH, precise identification by intraoperative ultrasonography (IOUS) was indispensable for the removal of multiple lesions located in the superficial or deep liver parenchyma and for confirmation of the hepatic vasculature. Parenchymal transection was performed using the cavitron ultrasonic surgical aspirator (CUSA) under intermittent pedicle clamping as Pringle maneuver. In order to shorten the time of Pringle maneuver and to reduce bleeding, if possible, some parts of liver transection could be undergone without the Pringle maneuver. To ensure efficient and certain removal of the lesions, a winding transection line could be set, resulting in the formation of a polymorphic curved cut-surface instead of a flat plane. And intrahepatic vessels thicker than 1–2 mm were ligated or clipped. Operative management for cases where a hepatic vein was adhered metastatic lesions was based on the IOUS findings and preoperative images. The hepatic vein could then be detached from the adjacent tumor at just the outer layer of the vessel by meticulous dissection with Metzenbaum scissors or CUSA. However, in cases where the tumor was adhered a large area of the hepatic vein or if deformities and/or stenotic changes were present, complete resection of the hepatic vein was required. The hepatic vein was not reconstructed in cases where other collateral veins, such as an inferior right hepatic vein or an adequate intrahepatic communicating vein, were present as documented in the preoperative imaging and/or IOUS [26].

**Fig. 3:**
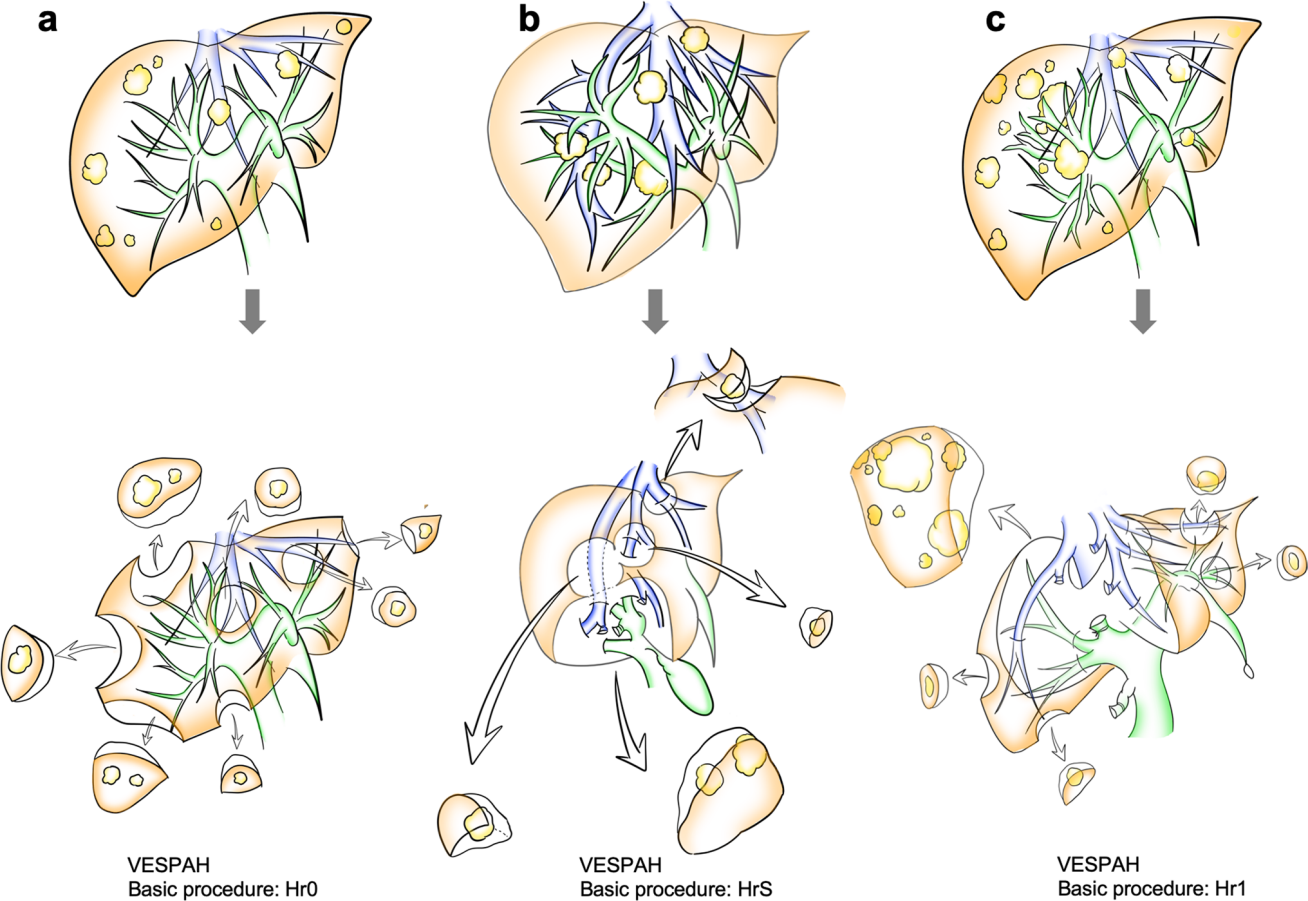
The various procedures of vessel-skeletonized parenchyma-sparing hepatectomy. **a** Multiple Hr0 (non-anatomical partial resections). **b** HrS (minimal anatomical segmentectomy) combined with multiple Hr0. **c** Hr1 (minimal anatomical sectionectomy) combined with multiple Hr0

**Fig. 4:**
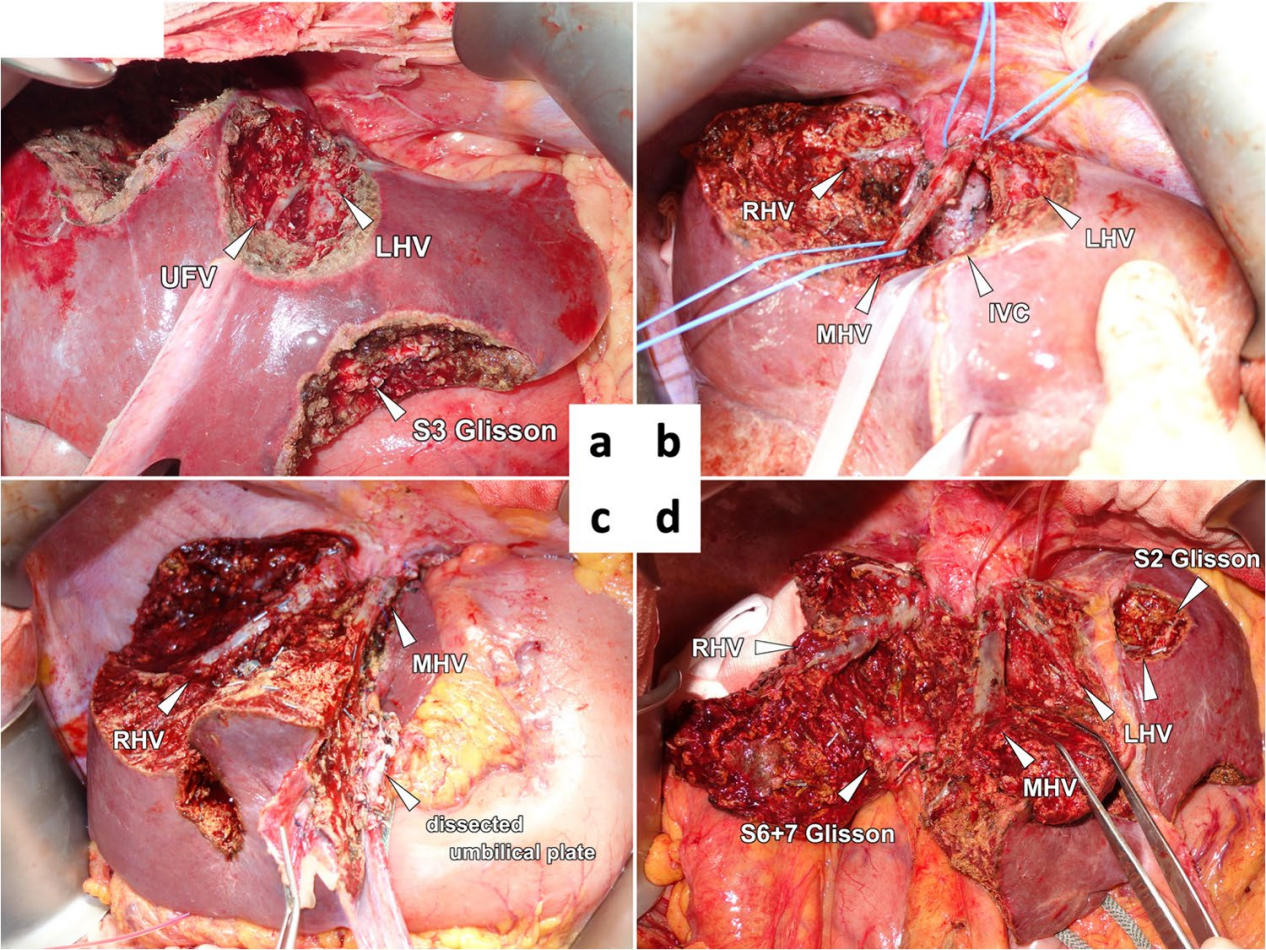
Examples of the basic procedures for vessel-skeletonized parenchyma-sparing hepatectomy. **a** Representative image from a patient who underwent the Hr0 procedure. The basic Hr0 procedure comprised 8 resection areas. Tumors in the deep parenchyma were completely removed via navigation of the skeletonized intrahepatic vessels. The total number of resected colorectal liver metastases was 8. **b** Representative image from a patient who underwent the HrS procedure. The basic HrS procedure (segmentectomy 1) was extended to the ventral liver. The total number of resected colorectal liver metastases was 8. **c** Representative image from a patient who underwent the Hr1 procedure. The anatomical segmentectomy 8 and partial resection were added to the basic Hr1 procedure (left lateral section) with nonanatomical extension to segment 4. Half of the umbilical plate was dissected. The total number of resected colorectal liver metastases was 27 (Supplementary Figure 1, schematic of the surgical procedures). **d** Representative image from a patient who underwent the Hr1 procedure. The basic Hr1 procedure (anterior section) was nonanatomically extended to segments 4, 6, and 7. Four partial resections were combined. Three hepatic vein trunks and the Glissonean pedicles were skeletonized on the cut surface of the liver. The total number of resected colorectal liver metastases was 53 (additional information is summarized in Supplementary Figure 2. Actual surgical procedure was uploaded in Supplementary VIDEO file). IVC, inferior vena cava; LHV, left hepatic vein; MHV, middle hepatic vein; RHV, right hepatic vein; UFV, umbilical fissure vein

The degree of liver transection depended on the type of VESPAH procedure performed (Figure 3). First, in the basic procedure that involved wedge or partial resection of the liver (Hr0), multiple resections, similar to cherry-picking, were performed (Figure 3a) [27]. An example of such case was presented in Figure 4a. In the transected area of the Hr0 procedure, Glissonean pedicles and/or hepatic veins were sometimes widely exposed as a result of detaching the tumor from these structures or of vessel navigation to reach lesions in the deep parenchyma.

Second, in the basic procedure that involved anatomical segmentectomy of the liver (HrS), the Glissonean pedicles invaded by the tumors were sacrificed to minimize the degree of anatomical resection. Each Glissonean pedicle was transected via an extrafascial and transfissural approach with major liver transection [28]. This transection area was determined by staining an anatomical segment via ultrasound-guided puncture and the injection of dye [29]. Thus, in the HrS procedure, several lesions were combined adjacently or separately (Figure 3b). In some special cases, resection of the entire caudate lobe (S1) extending to the ventral liver was performed (An example of such case was presented in Figure 4b).

Finally, in the basic procedure of sectionectomy of the liver (Hr1), two adjacent liver segments were removed. The Glissonean pedicle was ligated and transected via an extrahepatic approach without liver transection [28]. After the demarcation line was confirmed, a transaction area that included several colorectal liver metastases close to the border was determined (Figure 3c). In cases where the colorectal liver metastatic lesions were in contact with an umbilical plate and a middle hepatic vein, anatomical left lateral section of via an intrafascial approach with extension to upper segment 4 and detachment from the middle hepatic vein were included in the VESPAH procedure. Additional Hr0 and/or HrS procedures were added as necessary (An example of such case was presented in Figure 4c). Additionally, for numerous lesions scattered around the anterior sector, anterior sectionectomy was extended at the polymorphic transection line to the middle and posterior sectors with tumor detachment from all three hepatic vein trunks (An example of such case was presented in Figure 4d and Supplementary VIDEO 1. If necessary, Hr0 procedures and/or minimized HrS were combined with these basic VASPAH procedures of HrS and Hr1.

Concerning about perioperative management, quality of liver parenchyma is assessed by liver function test, Indocyanine green (ICG) clearance test, and 99mTc-GSA scintigraphy. And surgical plan is simulated by volumetric software, prior to surgery. It should also be noted that VESPAH increases the risk of bile leak due to the atypical extensive liver cut surface and the skeletonized Glissonean pedicle. Therefore, the Glissonean branch near the hilar region is treated thoroughly and carefully. In cases where the Glisson sheath near the hilum is extensively exposed on the liver cut surface, and leak test are performed routinely. The leak-point is repaired by suturing or ligation, and if there is still a tendency to leak, a C-tube is placed to decompress the biliary tract. As for bile leak, our policy is to wait for spontaneous healing with Drain replacement, but Endoscopic retrograde biliary drainage (ERBD) and reoperation are also considered for Grade B/C.

### Outcomes, follow-up, and treatment for recurrence

Overall survival (OS) was calculated from the date of the first hepatectomy for colorectal liver metastases to the date of death or last follow-up (for censored patients). Recurrence-free survival (RFS) was calculated from the date of the first hepatectomy to the date of the first documentation of local, regional, or distant relapse or the appearance of a second primary lesion, as determined by routine computed tomography scans, magnetic resonance imaging scans, or both, performed every 2–3 months. In principle, adjuvant chemotherapy was introduced as soon as possible, following the recovery of liver function. Not only hepatic recurrence, but also extra-hepatic metastatic recurrence like lung and lymph node metastasis were treated with surgical resection whenever possible. And early recurrences are treated with repeat resection after a period of 6–12 months, including chemotherapy.

### Statistical analysis

Statistical analyses were performed using JMP Pro software version 14.3.0 (SAS Institute, Inc., Cary, NC, USA). Continuous variables are expressed as the medians with ranges, and the Wilcoxon/Kruskal-Wallis test was used to compare continuous variables. Categorical variables were expressed as numbers and percentages and compared using the Chi-square test. OS and PFS were estimated by univariate analyses using the Kaplan–Meier method, expressed as medians, and compared with log-rank test. All reported *P*-values were calculated using two-sided tests, and *P*-values <0.05 were considered statistically significant.

### Ethics statement

This study conformed to the Declaration of Helsinki on Human Research Ethics standards and was approved by the Okayama University Hospital Institutional Ethics Board (number 2008-024). Since this study was retrospective in nature, there was no written informed consent from the investigated patients.

## Results

### Patient characteristics

Of the total 152 patients, 108 (71.1%) had multiple CRLMs. Among them, 86 patients (56.6%) had multiple bilobar CRLMs, and among these patients, 33 (21.7%) had multiple bilobar CRLMs (≥8 liver metastases). Of the 33 patients with 8 or more bilobar CRLMs, 20 patients (13.2%) were treated with the VESPAH procedure (VESPAH group), and the remaining 13 patients (8.6%) were treated with major hepatectomy (Major Hx group). The demographics of both groups are summarized in Table 1.

**Table 1:** Association of clinical factors between the VESPAH group and the Major Hx group

Variables	VESPAH (*n*=20)	Major hepatectomy (*n*=13)	*P*-value
Patients’ background			
Sex			
Male/female	13 (65%)/7 (35%)	4 (31%)/9 (69%)	0.801
Age			
Median (range)	65 (45–82)	57 (37–76)	0.076
Preoperative chemotherapy			
Yes	20 (100%)	13 (100%)	
Regimen			
Doublet-OX	11 (55%)	7 (54%)	0.094
Doublet-CPT	7 (35%)	1 (8%)	
Triplet	2 (10%)	3 (23%)	
Hepatic artery infusion	0	2 (15%)	
Use of target agent			
Yes	20 (100%)	8 (72%)	0.011
Anti-VEGF antibody	12 (60%)	5 (38%)	
Anti-EGFR antibody	8 (40%)	3 (23%)	
Tumor factor			
Timing of liver metastasis			
Synchronous	20 (100%)	13 (100%)	
Metachronous	0	0	
Sidedness of primary tumor			
Right	7 (35%)	3 (23%)	0.466
Left	13 (65%)	10 (77%)	
*Ras*-mutation			
Mutant	9 (45%)	5 (38%)	0.710
Existence of lymph node metastases in primary tumor			
Yes	20 (100%)	11 (85%)	0.070
Number of colorectal liver metastases			
Median (range)	13 (8–53)	10 (8–41)	0.511
8–10	9 (45%)	3 (23%)	0.434
11–19	7 (35%)	6 (46%)	
≥ 20	4 (20%)	4 (31%)	
Size of colorectal liver metastasis (cm)			
Median diameter of the largest lesion (range)	4.1 (1.2–12)	5.1 (2.5–16)	0.125
≤ 5 cm	11 (55%)	6 (46%)	0.619
> 5 cm	9 (45%)	7 (54%)	
Number of tumor-located segments			0.019
Median (range)	6 (3–8)	4 (3–7)	
Major hepatic vein contact			0.345
Yes	14 (70%)	7 (54%)	
Extra-hepatic metastatic lesion (at hepatectomy)			
Lung	2 (10%)	4 (33%)	
Para-aortic lymph node	2 (10%)	1 (8%)	0.120
Ovary		1 (8%)	
Preoperative risk score			
Beppu’s nomogram			
Median (range)	19 (15–25)	21 (15-25)	0.507
15–20	9 (45%)	6 (46%)	0.619
21–25	11 (55%)	7 (54%)	
GAME score			
Median score (range)	5 (3–6)	4 (3–7)	0.969
Medium-risk (GAME score 3)	1 (5%)	4 (31%)	0.043
High-risk (GAME score ≥ 4)	19 (95%)	9 (69%)	
Surgical procedure			
Basic procedure-Hr0	3 (15%)	0	
Total number of Hr0 procedures	8 (6–14)*		
Resected tumors	9 (8–16)*		
Basic procedure-HrS	8 (40%)		
Total number of combined Hr0 procedures	4 (1–7)*		
Resected tumors	9 (8–18)*		
Basic procedure-Hr1	9 (45%)		
Total number of combined Hr0 procedures	4 (3–5)*		
Resected tumors	13 (8–53)*		
Basic procedure-Hr2	0	10 (77%)	
Total number of combined Hr0 procedures		1 (1–5)*	
Resected tumors		11 (8–41)*	
Basic procedure-Hr3	0	3 (23%)	
Total number of combined Hr0 procedures		0	
Resected tumors		10 (9–10)*	
Surgical outcome			
Mean volume of blood loss (ml)	400 (60–1,755)	335 (120–1,600)	0.860
Total time of the Pringle maneuver (min.)	84 (37–129) *	38 (15–73)*	0.001
Total number of the Pringle maneuver	5 (3–11)	3 (2–5)	0.019
Resection margin: R0/R1-Par/R1-Vasc	9 (45%)/3 (15%)/8 (40%)	12 (92%)/1 (8%)/0	0.015
Overall complications: > grade II†	6 (30%)	4 (31%)	0.962
Bile leakage: grade A/B/C††	3 (15%)/4 (20%)/0	1 (8%)/3 (23%)/0	0.817
PHH: grade A/B/C††	1 (5%)/0/0	2 (15%)/1 (8%)/0	0.274
PHLF: grade A/B/C††	2 (10%)/1 (5%)/0	0/4 (31%)/1 (8%)	0.048
Postoperative outcome			
Induction of adjuvant chemotherapy within 2 post operation months			0.002
Yes	18 (90%)	5 (38%)	
Recurrent site and type after hepatectomy			
No recurrence/intrahepatic/extrahepatic/both	3 (15%)/8 (40%)/3 (15%)/6 (30%)	4 (31%)/6 (46%)/2 (15%)/1 (8%)	0.416
Additional hepatic/hepatic local recurrence**	13 (93%)/1 (7%)	6 (86%)/1 (14%)	0.599
Treatment for hepatic recurrence**			0.147
Chemotherapy alone	2 (14%)	3 (43%)	
Repeat hepatectomy + chemotherapy	12 (86%)	4 (57%)	
Total number of hepatectomy			0.060
Hepatectomy-once	8 (40%)	9 (69%)	
Hepatectomy-twice	5 (25%)	3 (23%)	
Hepatectomy-thrice	7 (35%)	0	
Hepatectomy-four times	0	1 (8%)	

*Abbreviations*: *CPT*, irinotecan; *Hr0*, wedge or partial resection; *Hr1*, sectionectomy; *Hr2*, hemihepatectomy; *Hr3*, trisectionectomy; *HrS*, segmentectomy; *OX*, oxaliplatin; *PHH*, posthepatectomy hemorrhage; *PHLF*, posthepatectomy liver failure

^*^Median (range) , **In intrahepatic recurrence

^†^Clavien-Dindo classification, ††Definition of International Study Group of Liver Surgery (ISGLS)

All patients in both groups were treated with preoperative chemotherapy before hepatectomy. During preoperative chemotherapy treatment, targeted agents were more frequently used in the VESPAH group (*n*=20 [100%]) than in the Major Hx group (*n*=8 [72%]; *P*=0.011).

Regarding the primary tumors, the 33 patients analyzed in this study possessed synchronous metastatic lesions. There was no difference in the location of the primary tumors, the frequency of *RAS* mutations, or existence of lymph node metastases in primary tumor between the VESPAH group and the Major Hx group.

Concerning the CRLMs, there were no differences in the number or size of CRLMs between the two groups. In contrast, the number of segments with metastatic lesions was significantly higher in the VESPAH group than in the Major Hx group (the median number of segments with tumors was 6 in the VESPAH group and 4 in the Major Hx group, *P*=0.019).

We evaluated preoperative risk scores that have been reported to predict clinical outcomes after hepatectomy, e.g., Beppu’s nomogram and the GAME score. According to Beppu’s nomogram, there was no difference between the groups. However, although there was no difference in the median GAME score between the two groups, patients who were categorized as high risk (i.e., GAME score ≥ 4) were more frequently observed in the VESPAH group than in the Major Hx group (*P*=0.043).

### Resections and removal of metastases

CRLMs in the VESPAH group were removed by partial resection (Hr0, *n*=3), minimal anatomical segmentectomy (HrS, *n*=8), or minimal anatomical sectionectomy (Hr1, *n*=9). In contrast, metastases in the Major Hx group were removed by Major Hx, i.e., right/left hemihepatectomy (Hr2, *n*=10) or right/left trisectionectomy (Hr3, *n*=3). In the VESPAH group, the patients who underwent the Hr0 procedure had a median of 8 resection areas (range, 6–14), and a median of 9 CRLMs (range, 8–16) were removed. Of the patients who were treated with the HrS or Hr1 procedure, additional Hr0 procedures were combined with basic HrS or Hr1. The patients treated with the HrS procedure underwent a median number of 4 Hr0 resections (range, 1–7), and a median of 9 CRLMs (range, 8–18) were removed. The Hr1 patients underwent a median of 4 Hr0 resections (range, 3–5), and a median of 13 CRLMs (range, 8–53) were removed. In the Major Hx group, the patients treated with the Hr2 procedure underwent a median of 1 Hr0 resection (range, 1–5), and a median of 11 CRLMs (range, 8–41) were removed. The patients who underwent the Hr3 procedure were not treated in combination with Hr0 resection, and a median of 10 CRLMs (range, 9–10) were removed.

### Surgical and oncological outcomes

As for intermittent vascular occlusion during hepatectomy, the VESPAH group required the Pringle maneuver more frequently and for longer time than the Major Hx group (the median time was 84 min in the VESPAH and 38 min in the Major Hx group, *P*=0.001). Concerning intra- and perioperative outcomes, there was no operative mortality, and no difference was observed in the mean volume of blood loss, occurrence of posthepatectomy hemorrhage or bile leakage between the two groups; however, the incidence of post hepatectomy liver failure (PHLF) grade B/C was less frequent in the VESPAH group than in the Major Hx group (incidence of PHLF Grade B/C was in 5% in the VESPAH group and 38% in the Major Hx group, *P*=0.048). After the patients physically recovered and regained remnant liver function, 18 patients (90%) in the VESPAH group received adjuvant chemotherapy within 2 months after surgery, whereas 5 patients (38%) in the Major Hx group received adjuvant chemotherapy within the same time period (*P*=0.002). The VESPAH group resulted in significantly higher rate of R1 surgery than the Major Hx group (rates of R1-Par/R1-Vasc was 15%/40% in the VESPAH group, and 8%/0 in the Major Hx group, *P*=0.015). However, the recurrence rate and type after hepatectomy were not different between the two groups. Local hepatic recurrence was comparable between two groups (rate of local recurrence was 7.1% in the VESPAH group, and 14.3% in the Major Hx group, *P*=0.599). According to surgical margin status, hepatic local recurrence was seen in 8.3% of R0, in 33% of R1-parc, and none of R1-vasc (*P*=0.269; Supp. Figure 3). Although there was no significant difference, the median number of repeat hepatectomy for intrahepatic recurrence during the patients’ lifetimes tended to favor the VESPAH group (*P*=0.06).

**Fig. 5:**
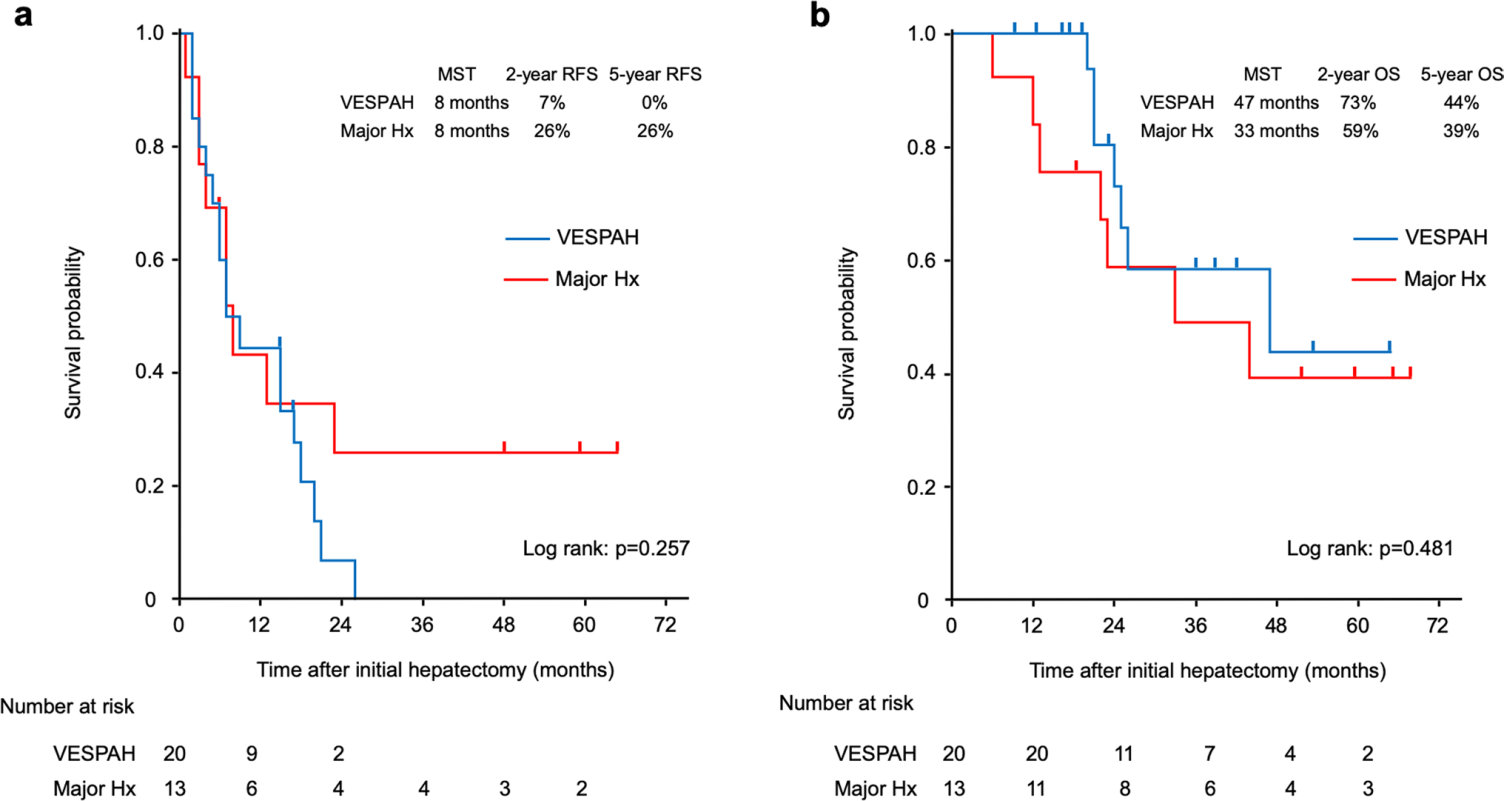
Kaplan-Meier curves of recurrence-free survival (**a**) and overall survival (**b**) after the first hepatectomy

The median RFS after hepatectomy in the VESPAH group was almost the same as that in the Major Hx group (8 months in the VESPAH and 8 months in the Major Hx group, *P*= 0.257; Figure 5a). Furthermore, there was no significant difference in median OS after hepatectomy between the two groups; the median OS was 47 months in the VESPAH group and 33 months in the Major Hx group (*P*=0.481; Figure 5b). Moreover, the 5-year OS rate was 43.8% in the VESPAH group and 39.1% in the Major Hx group.

## Discussion

Herein, we presented a surgical procedure, VESPAH, that is based on the surgical philosophy of parenchyma-sparing hepatectomy. The concept and surgical management of parenchyma-sparing hepatectomy have been well summarized by Alvarez FA et al [30]. The essential findings are that VESPAH allowed the removal of all colorectal liver metastases with maximum preservation of intrahepatic structures, thus may leading to be able to most of patients receive adjuvant chemotherapy within 2 months after hepatectomy and repeat hepatectomies during the patients’ lifetimes.

To ensure a large future remnant liver volume after hepatectomy, portal embolization and planned TSH have been developed in addition to parenchyma-sparing hepatectomy. However, the main concern with TSH is the drop-out rate, as many patients cannot progress to second-stage hepatectomy due to difficulties achieving disease control during the interval between the scheduled hepatectomies. Generally, conventional TSH requires a 4-week interval between the first and second hepatectomies to increase the future remnant liver volume. This 4-week interval without tumor control allows for rapid cancer growth in some cases. The median drop-out rate of TSH has been reported to be 23% (range 0–36%) [12]. The following risk factors for TSH drop out have been reported: a maximum tumor diameter > 40 mm, >12 chemotherapy cycles, progressive disease during first-line chemotherapy, and carcinoembryonic antigen levels >30 ng/ml [31].

ALPPS has been established to overcome the limitations of TSH by shortening the interval between scheduled hepatectomies [9]. Although ALPPS might be a valid and promising option, the surgical risks should be noted. The perioperative mortality rate of ALPPS has been reported to be 4.9–9% [13, 32, 33], which is mainly due to liver failure caused by the divergence of morphological and functional liver regeneration, as well as infections caused by bile leakage and liver infarction. Although ALPPS has a shorter interval than TSH between scheduled hepatectomies, during which there is no tumor control, the interval is still not short enough to prevent cancer progression in the residual liver. Indeed, molecules that induce regeneration of the remnant liver volume, e.g., hepatocyte growth factor (HGF), epidermal growth factor, and insulin-like growth factor, are increased in the serum after a first-scheduled hepatectomy, but these factors also stimulate the growth of residual colorectal tumors [34–36]. In light of the problems associated with scheduled two-stage hepatectomies, one-stage hepatectomy is ideal [37, 38]. By applying VESPAH, we have a possibility for approaching the ideal of one-stage hepatectomy to remove bilobar multiple CRLMs.

As our analyzed patients had 8 or more bilobar CRLMs, they were almost classified within the high-risk category by Beppu’s nomogram and the GAME score. Beppu’s nomogram predicted a 2-year RFS rate and 5-year OS rate of less than 15% and 11%, respectively, in our cohort [18]. Indeed, the 2-year RFS rate was 7% in the VESPAH group and 26% in the Major Hx group. However, the 5-year OS rate reached 44% in the VESPAH group and 39% in the Major Hx group, which was beyond that predicted by GAME score. Although the recurrence outcome was in accordance with this prediction in the both groups, patients with recurrence survived for a longer period after surgery in the both group in this study (the median OS in the VESPAH group was 47months and it in the Major Hx group was 33 months, *P*=0.481). This favorable outcome could be achieved with multimodal treatment, including repeat hepatectomy and chemotherapy. Our results demonstrate an advantage of VESPAH in that 90% of patients who underwent VESPAH were able to receive adjuvant chemotherapy within 2 months after hepatectomy. Regarding tumor number, numerous CRLM may not be an optimal surgical indication. In this analysis, all patients with CRLM ≥8 who were eligible for surgery were those who had received preoperative chemotherapy and had a good response. Median OS/5-year OS rate were 78 months/66.7% in CRLM ≥ 20 (*n*=8), and 44 months/35.2% in CRLM < 20 (*n*=25), respectively (data not shown). Although this result would be limited because of a small study, the number of tumors alone could not be used to determine the indications for surgery, and that it is acceptable to explore the possibility of surgery in multimodal treatment by taking other factors into consideration.

We found that VESPAH reduced the resection volume and to preserve the inflow and outflow of the Glissonean pedicle vasculature and major hepatic veins to maintain remnant liver function and promote liver regeneration. The main concern with VESPAH is whether the detachment of tumors from intrahepatic structures is oncologically permissible. Preserving hepatic veins is an essential for preserving the inflow and outflow of the Glissonean pedicle vasculature and major hepatic veins to maintain remnant liver function. Tumors are often detachable from hepatic veins unless adhesions with deformities are present. Preserving of hepatic veins by venous detachment from metastatic lesion has been associated with a low local recurrence rate in patients who respond to chemotherapy, making it worthwhile to perform dissection [24, 25, 39]. Recently, Laennec’s capsule has been established as a novel comprehensive anatomical structure that can contribute to precise liver surgery, particularly for aiding in tumor detachment [40]. Transection of cardiac Laennec’s capsule which covered main hepatic vein’s wall has been reported as the “inter-Laennec approach” [41]. VESPAH also adopts this inter-Laennec approach to transect colorectal liver metastases which are in contact with major hepatic vein or the Glissonean sheaths. This precise anatomical structure could enable us feasible and certain tumor detachment with a negative surgical margin. Indeed, in this study, no patients in the VESPAH group experienced regional recurrence at sites where the tumors were detached from major hepatic veins. And VESPAH is a concept of surgical technique, so of course it can be applied even if the number of tumors is 7 or less.

In conclusion, in this report, we present a surgical procedure and the outcomes of VESPAH for the removal of all CRLMs with maximal preservation of intrahepatic structures via clear surgical navigation achieved through hepatic vessel skeletonization. The limitations of this study are its retrospective nature and the small number of analyzed cases. And of 10 patients who underwent hemihepatectomy in the Major Hx group, it is possible that VESPAH was indicated in some cases where there was no undetachable invasion of the primary Glisson and/or no more than two hepatic veins. In fact, after the introduction of VESPAH, there were a couple of cases in which major hepatectomy had to be performed in tumor conditions. Perhaps the effect of VESPAH derived by this analysis may be biased results.

However, we found that most of patients who underwent VESPAH were able to recover without severe PHLF and to receive adjuvant chemotherapy within 2 months after hepatectomy. This tolerance for chemotherapy might become more important to improve the clinical outcomes of CRC patients with advanced CRLMs. The VESPAH is a surgical approach that focuses on exactly this point in the multidisciplinary treatment of multiple liver metastases. It seems impossible to cure bilobar multiple CRLMs with a single hepatectomy by complete resection including latent lesions and disappearing lesions. Rather than increasing the resection volume with unnecessary or uncertain blind hepatectomy, the main focus should be on the prompt introduction and continuity of postoperative chemotherapy, leading to repeat hepatectomy. However, in cases with multiple bilobar CRLMs which showed extensive invasion to the main Glissonean pedicle, VESPAH would not be indicated. In such a situation, at least hemihepatectomy and multisite resections for remnant lobe would be inevitable. If the remaining liver volume could not be safely secured, portal vein embolization and two-stage approach by TSH or ALPPS could be alternative options. And perhaps it may not be worthwhile to compare one-stage removal by VESPHA with two-stage approach by TSH or ALPPS. Because the concept of VESPAH as a surgical technique could be applied in the first-stage surgery in TSH and ALPPS. As a result, it has the potential to further improve the resection rate of unresectable CRLM. VESPAH may become the evening star “Vesper” that illuminates the patient’s wish to cure, along with the possibility of resection for all advanced CRLMs.

## Supplementary Information

Below is the link to the electronic supplementary material.

Supplementary Figure 1 Surgical Schematic of a case who treated by VESPAH (basic procedure: Hr1) (PNG 2157 kb)

Supplementary file1 (TIFF 8792 kb)

Supplementary Figure 2 Multiple colorectal liver metastases (53 liver metastases, including the lesion with MHV/LHV involvement). Right anterior sectionectomy was nonanatomically extended in a winding manner to segments 4, 6, and 7. Finally, the detached MHV/LHV trunk, RHV trunk, and Glissonean pedicles for segments 7 and 6 were skeletonized and exposed on the cut surface of the liver. Subsequently, four partial resections were additionally performed (operation time: 7 h 50 min, total blood loss: 660 ml)**.** LHV. left hepatic vein; MHV. middle hepatic vein; RHV. right hepatic vein (PNG 7583 kb)

Supplementary file2 (TIFF 8792 kb)

Supplementary Figure 3 **a)** Proportion of resection margin status (n=33)**, b)** Pattern of intrahepatic recurrence (n=21)**, c)** Correlation between pattern of intrahepatic recurrence and resection margin status (n=21) (PNG 199 kb)

Supplementary file3 (TIFF 8792 kb)

Supplementary file4 (MP4 98644 kb)
