# Supplementary figures and images for: Technique of vessel-skeletonized parenchyma-sparing hepatectomy for the oncological treatment of bilobar colorectal liver metastases
Source: Langenbecks Arch Surg. 2021 Nov 27;407(2):685–97. doi: 10.1007/s00423-021-02373-9 (PMC8933371; doi:10.1007/s00423-021-02373-9)

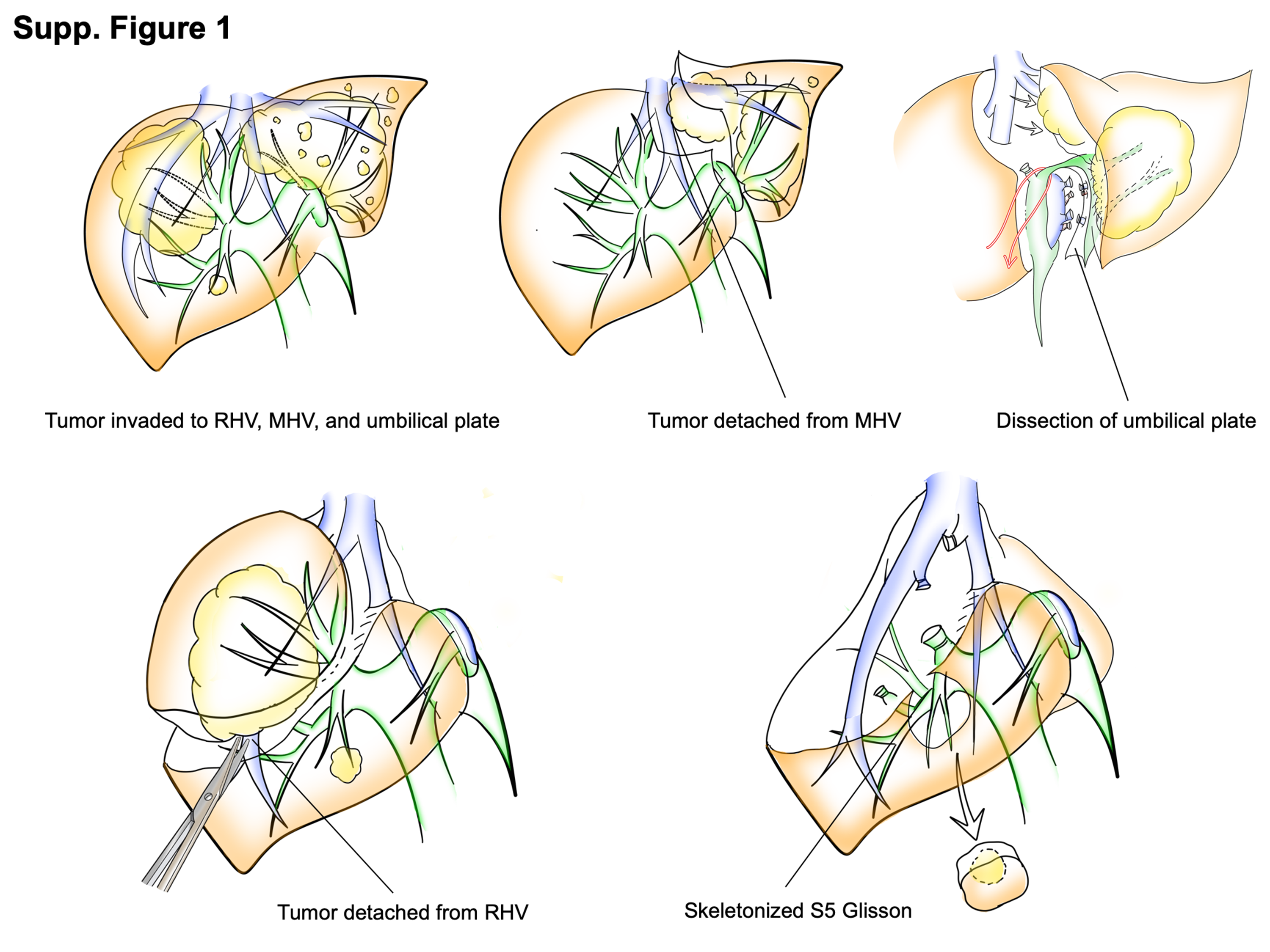

Supplement: Supplementary file 1 — Surgical Schematic of a case who treated by VESPAH (basic procedure: Hr1) (PNG 2157 kb) [file 423_2021_2373_Fig6_ESM.png]

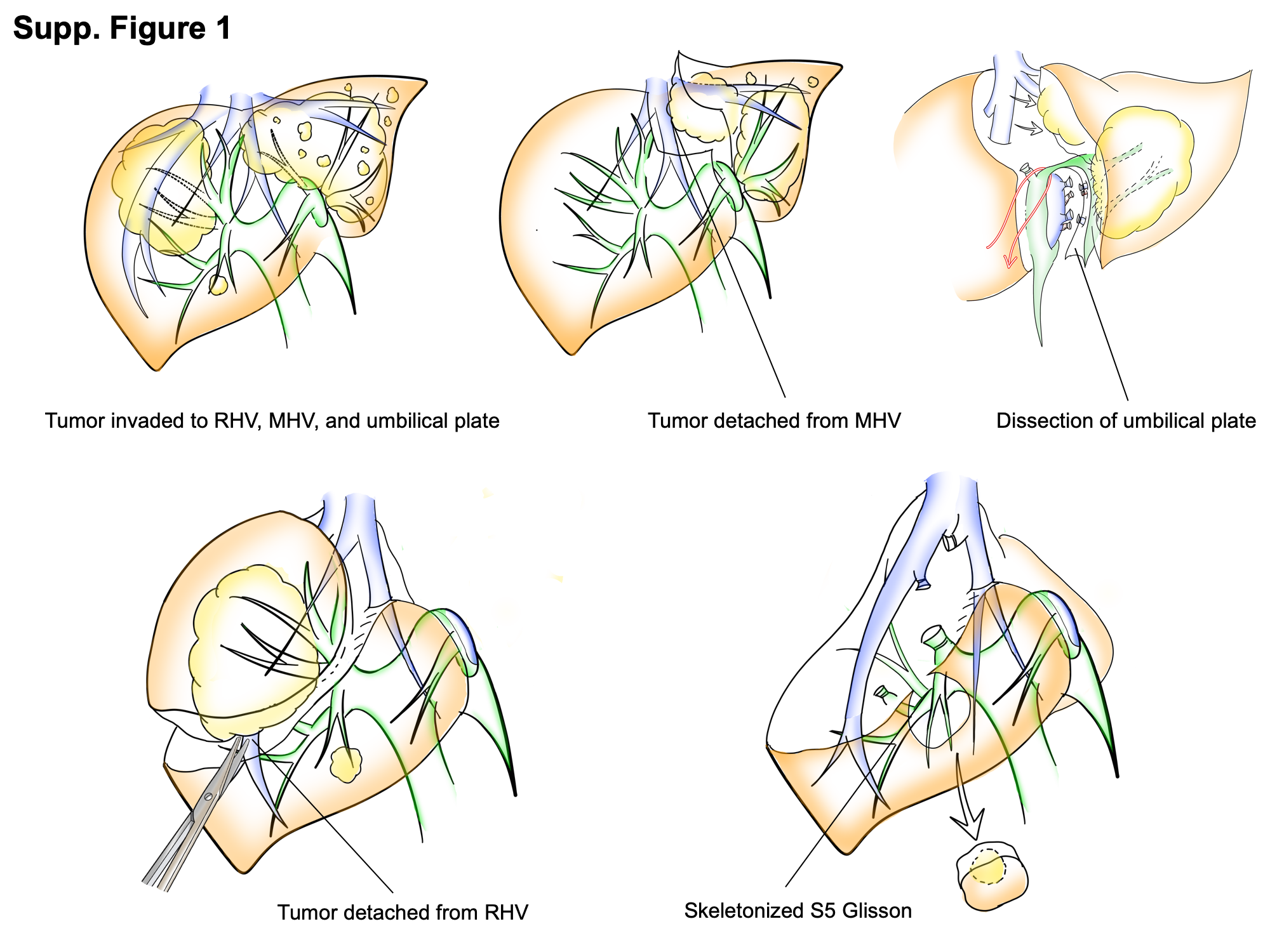

Supplement: Supplementary file 2 — Supplementary file1 (TIFF 8792 kb) [file 423_2021_2373_MOESM1_ESM.tiff]

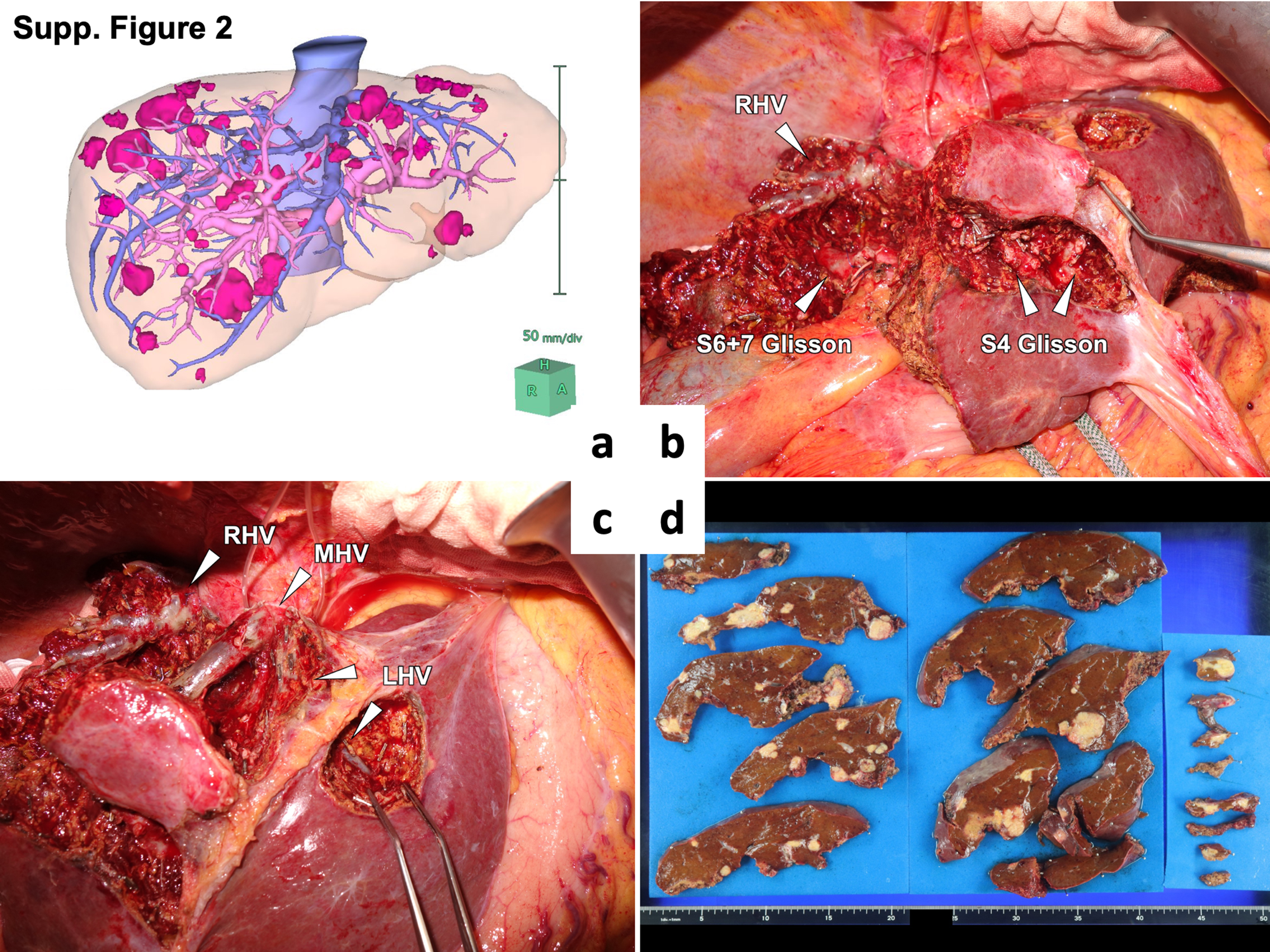

Supplement: Supplementary file 3 — Multiple colorectal liver metastases (53 liver metastases, including the lesion with MHV/LHV involvement). Right anterior sectionectomy was nonanatomically extended in a winding manner to segments 4, 6, and 7. Finally, the detached MHV/LHV trunk, RHV trunk, and Glissonean pedicles for segments 7 and 6 were skeletonized and exposed on the cut surface of the liver. Subsequently, four partial resections were additionally performed (operation time: 7 h 50 min, total blood loss: 660 ml). LHV. left hepatic vein; MHV. middle hepatic vein; RHV. right hepatic vein (PNG 7583 kb) [file 423_2021_2373_Fig7_ESM.png]

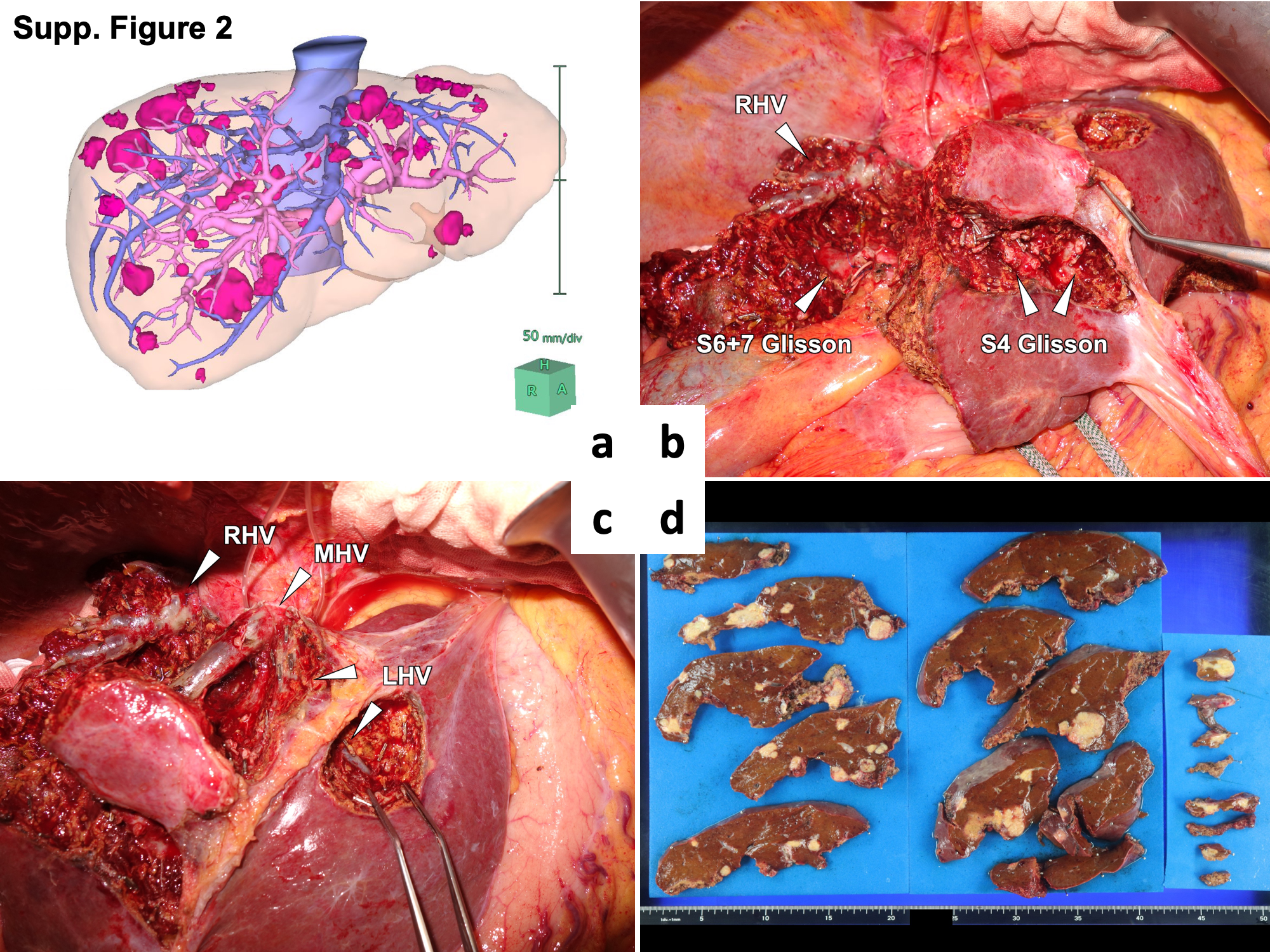

Supplement: Supplementary file 4 — Supplementary file2 (TIFF 8792 kb) [file 423_2021_2373_MOESM2_ESM.tiff]

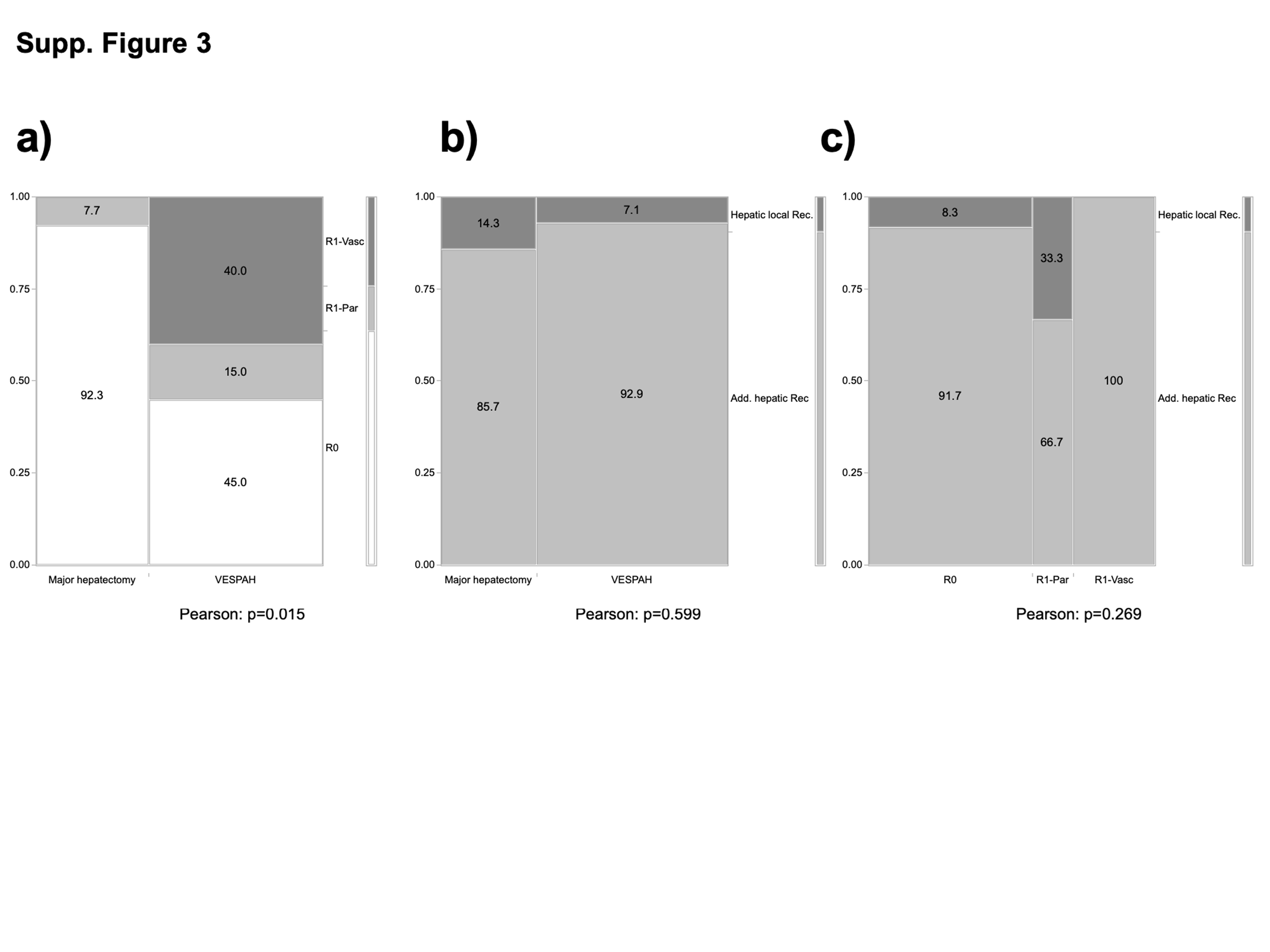

Supplement: Supplementary file 5 — a) Proportion of resection margin status (n=33), b) Pattern of intrahepatic recurrence (n=21), c) Correlation between pattern of intrahepatic recurrence and resection margin status (n=21) (PNG 199 kb) [file 423_2021_2373_Fig8_ESM.png]

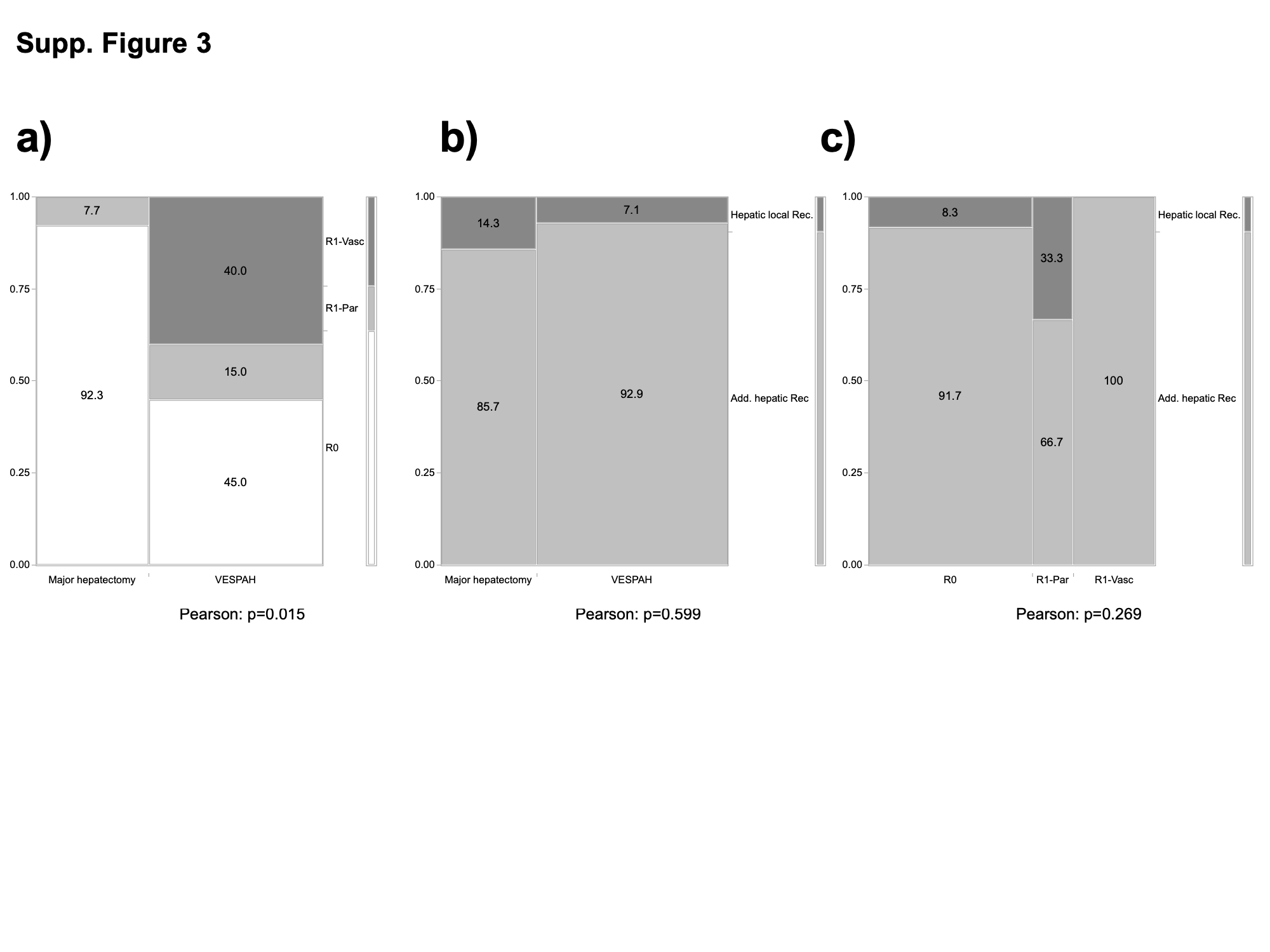

Supplement: Supplementary file 6 — Supplementary file3 (TIFF 8792 kb) [file 423_2021_2373_MOESM3_ESM.tiff]
